# Contribution of Cholesterol and Oxysterols in the Physiopathology of Cataract: Implication for the Development of Pharmacological Treatments

**DOI:** 10.1155/2011/471947

**Published:** 2011-04-04

**Authors:** Anne Vejux, Mohammad Samadi, Gérard Lizard

**Affiliations:** ^1^Inserm-CIT 808, CHU de Besançon, 25030 Besançon, France; ^2^Equipe Biochimie Métabolique et Nutritionnelle Centre de Recherche INSERM 866 (Lipides, Nutrition, Cancer), Faculté des Sciences Gabriel, Université de Bourgogne, 6 Boulevard Gabriel, 21000 Dijon, France; ^3^LCME/Département de Chimie, Université Paul Verlaine-Metz, 57012 Metz, France

## Abstract

The development of cataract is associated with some lipid changes in human lens fibers, especially with increased accumulation and redistribution of cholesterol inside these cells. Some direct and indirect lines of evidence, also suggest an involvement of cholesterol oxide derivatives (also named oxysterols) in the development of cataract. Oxysterol formation can result either from nonenzymatic or enzymatic processes, and some oxysterols can induce a wide range of cytotoxic effects (overproduction of reactive oxygen species (ROS); phospholipidosis) which might contribute to the initiation and progression of cataract. Thus, the conception of molecules capable of regulating cholesterol homeostasia and oxysterol levels in human lens fibers can have some interests and constitute an alternative to surgery at least at early stages of the disease.

## 1. Cholesterol Oxidation Products (Oxysterols): Definition and Biosynthesis

Oxysterols are 27-carbon-atom cholesterol oxidation products [1]. They can be produced endogenously by enzymatic reactions or by autoxidation. They also can be provided by food [1]. The enzymatic pathways can form both B-ring and side-chain hydroxylated oxysterols depending on the enzyme and the tissue, while the nonenzymatic pathways form mainly B-ring oxysterols. 

By the enzymatic pathway, oxysterols can be generated by a wide number of CYP450 enzymes [2]. Some of them are tissue specific. Thus, CYP46A1 (or 24-hydroxylase) leading to the formation of 24-hydroxycholesterol has been identified in the brain [3] and retina [4]. CYP7A1 which catalyzes the formation of 7*α*-hydroxycholesterol is present in the liver, and involved in bile acid synthesis [5]. At the opposite, some other CYP450 enzymes are widely expressed. Thus, CYP27A1 (or 27-hydroxylase), which catalyzes the addition of a hydroxyl group on cholesterol to produce 27-hydroxycholesterol, is found in most tissues [6]. Cholesterol 25-hydroxylase, leading to the formation of 25-hydroxycholesterol, is a nonheme iron protein enzyme, also present in many tissues [7].

**Figure 1:**
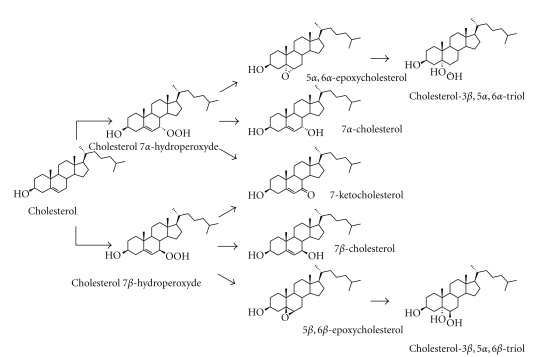
Cholesterol autoxidation. Autoxidation of cholesterol can generate 7*α*- or 7*β*-hydroperoxyde, 7*α*- or 7*β*-hydroxycholesterol, 7-ketocholesterol, 5*α*, 6*α*- or 5*β*, 6*β*-epoxycholesterol, as well as cholesterol 3*β*, 5*α*, 6*β* triol or cholesterol 3*β*, 5*α*, 6*α* triol depending on pH conditions.

Oxysterols can also be generated within tissues by nonenzymatic oxidative reactions involving different chemical and/or physical agents: reactive oxygen species (ROS), ozone, ultra violet light, metal ions, ferritin, and/or other iron-carrying proteins, and so forth. These autoxidation processes generate 7*α*- or 7*β*-hydroperoxyde, 7*α*- or 7*β*-hydroxycholesterol, and 7-ketocholesterol, 5*α*, 6*α*- or 5*β*, 6*β*-epoxycholesterol, as well as cholesterol 3*β*, 5*α*, 6*β* triol or cholesterol 3*β*, 5*α*, 6*α* triol depending on pH conditions (Figure 1) [8–10]. In certain conditions, 7-ketocholesterol can be produced from 7*β*-hydroxycholesterol and vice versa by a converting enzyme [11]. 

Currently, in humans, the involvement of oxysterols is suspected in the Smith-Lemli-Opitz syndrome [12, 13], and in numerous eye diseases (age-related macular degeneration, diabetic retinopathy,) [14, 15], and could contribute to the development of cataract [16].

## 2. Cholesterol and Oxysterols: Which Roles in Cataract?

Cataract, which is a term referring to the clouding of the eye's natural lens, is the dominant cause of blindness worldwide [17]. This disease develops as early as the fourth of fifth decade of life in the crystalline lens of the eye or in its envelope, varying in degree from slight to complete opacity and obstructing the passage of light. Symptoms include blurred vision, glare, halos, dull colors, and cloudy vision. Whereas the most important factor in cataract formation is increasing age, it is well admitted that cataract formation is a multifactorial disease associated with additional factors such as smoking, diabetes, and excessive exposure to sunlight which are known to activate oxidative stress [17]. Currently, surgical intervention is the most frequent and efficient treatment to restore vision in patients with cataract. However, the cure for cataract surgery is not equally available to all, and the surgery which is available does not produce equal outcomes. In addition, readily available surgical services capable of delivering good vision rehabilitation are not always acceptable and accessible to all in need. Therefore, a better knowledge of the physiopathology of cataract is required in order to attempt to develop, if not curative, at least preventive treatments. 

The concept suggesting a possible involvement of lipids in human cataract is based on the description of lipoidal material in the crystalline lens reported by Berzelius in 1825 [18]. Since this early discovery, some investigators have studied the lenticular lipids leading to lens opacity. In 1965, by using one and two dimensional thin layer chromatography, Feldman GL and Felman LS show higher amounts of cholesterol, cephalins, lecithin, and shingomyelin in cataractous human lens when compared to normal lens, and they also show that cholesterol is constitutively present in large amount in normal lens [16]. Therefore, cholesterol representing approximately 40% of the total lipids of human lens fibers [19], intrinsic or extrinsic factors modifying its level and/or repartition, may alter optical lens properties. Some cholesterols can be present as crystals, which have been found in plasma membranes isolated from the lens [20, 21], and which may play functional roles in normal and pathological lens [22]. The formation of these crystals is related to the lipid composition of the lens, and seems to depend on the presence of sphingomyelin and dihydrosphingomyelin [23]. The part taken by cholesterol in the development of cataract is also supported by observations performed in various pathologies associated with defects in cholesterol metabolism. Thus, patients with Smith-Lemli-Opitz syndrome, mevalonic aciduria, or cerebrotendinous xanthomatosis characterized by mutations in enzymes of cholesterol metabolism (7-dehydrocholesterol reductase, mevanolate kinase, and CYP27A1, resp.) often develop cataract [24]. In addition, established models of rodent cataracts are based on treatment with inhibitors of cholesterol biosynthesis, and some statins can produce cataracts in dogs [24]. Moreover, with regards to oxidative damages, as the lipid lens composition is devoid of oxidizable polyunsaturated fatty acids, and as there is a high content of dihydrosphingomyelin that is less prone to oxidation, this particular lipid composition favors cholesterol autoxidation. Thus, as human lens membrane contains the highest cholesterol levels of any known biological membranes, and as human lens is continuously in a strong photoxidative environment, a chronic exposure to UV light, and ozone can lead to the formation of some cholesterol oxide derivatives (also named oxysterols) [25–30] which might contribute to disrupt cholesterol repartition and homeostasia in human lens fibers. Noteworthy, on human cataracts obtained by routine extracellular surgery, some oxysterols characterized by gas chromatography were identified (7*β*-hydroxycholesterol, 7-ketocholesterol, 5*α*, 6*α*-epoxycholestanol, 20*α*-hydroxycholesterol, and 25-hydroxycholesterol) whereas clear lens contained no detectable amounts of cholesterol oxides [31]. These data favor the hypothesis that oxysterols may be involved in cataract development. Moreoever, as 7-ketocholesterol has been described to modify Na/K ATPase activity [32], and intracellular lipid homeostasia [33], this oxysterol might constitute an important risk factor in the physiopathology of cataract. Indeed, it has been described that Na/K ATPase activity is fundamental to the maintenance of ionic concentration gradients and transparency of the lens [34], and that unusual lipid composition modify lens membrane fluidity [35].

Some indirect arguments also support potential involvement of cholesterol and oxidative stress, mainly able to favor the formation of oxysterols oxidized at C7 [11], in the development of cataract: decrease paraoxanase 1 activity and higher levels of oxidized LDL in diabetic and senile subjects suffering from cataract [36], low level of HDL cholesterol and high LDL : HDL ratios in dyslipidaemic subjects with lenticular opacities [37], low serum concentrations of the antioxidant vitamins alpha tocopherol and beta carotene in end age senile cataract [38], and significant decrease of glutathione reductase activity in patients with cortical cataract [39]. In addition, as some oxysterols are known to interact with cellular membranes [40–42] and to induce changes in cholesterol and phospholipids content [43, 44], they could also modify the distribution of cholesterol in human lens fibers to contribute to lens opacity [45–49].

## 3. Alternative Pharmacological Treatments to Cataract Surgery

Phacoemulsification developed by Kelman in 1967 [50] is nowadays the preferred technique in most types of cataract. It results in less postoperative inflammation and astigmatism, more rapid visual rehabilitation and, with modern foldable lenses, a lower incidence of capsulotomy than with the outdated extracapsular surgery [50]. However, whereas surgical treatment with intraocular lens implantation remains the only proven treatment, it is associated with significant cost and is not readily available especially in the developing countries where the prevalence of cataract is the highest [51]. Therefore, nonsurgical preventive actions have been proposed to interact at the level of altered lens metabolism: Aldose-Reductase inhibitors (to block the metabolic pathways of glucose responsible for diabetic vascular dysfunction); nonsteroidal anti-inflammatory drugs (as prophylactic anticataract agents); agents enhancing reduced glutathione levels; Vitamins (Vitamin C plays an important part in lens biology, both as an antioxidant, and as a UV filter); minerals (zinc, copper, selenium); antioxidants (carotenoids, curcumin, stobadine, etc), and herbal drugs [52, 53]. However, the long-term efficiency of these alternative pharmacological treatments of cataract is far to be established. Therefore, within this framework, the research for molecules that can act at the level of cholesterol or oxysterols metabolism and/or synthesis could be promising.

Currently, due to a better knowledge of the cholesterol-metabolic pathway and of its regulation through various tightly regulated cellular systems involving various nuclear receptors [54], some molecules capable of regulating cholesterol levels have been identified and are available [55–57]. It can therefore be envisaged to modulate cholesterol levels in various cells, including lens fiber cells. On the other hand, due to the improvement of biochemical methods of analyses, especially mass-chromatography, some oxysterols can be measured and identified in various biological samples which usually contain a 10^3^ fold excess of cholesterol [58–60]. As some oxysterols with specific structural motifs have been shown to inhibit cholesterol synthesis by interacting with proteins involved in regulation of transcription of genes encoding enzymes of the cholesterol synthesis pathway [61] and to be ligands of the liver X receptors (LXRs) [62, 63] acting as regulators of the expression of genes important for lipid homeostasis, a better knowledge of oxysterols-associated metabolic profiles has some interests in various pathologies resulting from lipid disorders, and could therefore have some pharmacological applications, especially for the treatment of cataract. 

Thus, at the opposite of micronutrient and vitamin supplementation which can contribute more or less efficiently to prevent the development of cataract, it is tempting to speculate that the development of drugs capable of acting on well-defined targets of cholesterol metabolism, and on enzymatic and/or nonenzymatic formation of oxysterols might be efficient to preserve cholesterol homeostasia and distribution in human lens fibers, and to control oxysterol formation and activities. Such drugs could therefore constitute an alternative to surgery at least at the early stage of the disease.

## 4. Conclusion

Thus, based on numerous data and comparatively to other degenerative diseases [64, 65], it is tempting to speculate that cholesterol and some oxysterols probably play important roles in the physiopathology of cataract. Therefore, molecules allowing to control cholesterol and oxysterol levels in the lens might have some interests to prevent cataract and constitute an alternative treatment to surgery, at least at early stages of the disease.
